# Comparison of denosumab and oral bisphosphonates for the treatment of glucocorticoid-induced osteoporosis: a systematic review and meta-analysis

**DOI:** 10.1186/s12891-022-05997-0

**Published:** 2022-11-29

**Authors:** Lianghai Jiang, Jian Dong, Jianwei Wei, Lantao Liu

**Affiliations:** 1 Department of Spinal Surgery, Qingdao Municipal Hospital, Qingdao, 266000 Shandong China; 2 Department of Orthopedics, Dianjiang People’s Hospital Of Chongqing, Chongqing, 408300 China

**Keywords:** Denosumab, Diphosphonates, Glucocorticoids, Osteoporosis, Bone density, Meta-analysis

## Abstract

**Background:**

Both denosumab and bisphosphonates have been demonstrated effective for glucocorticoid-induced osteoporosis. However, evidence-based medicine is still lacking to prove the clinical results between denosumab and bisphosphonates. This meta-analysis aims to compare the efficacy and safety between denosumab and oral bisphosphonates for the treatment of glucocorticoid-induced osteoporosis through evidence-based medicine.

**Methods:**

MEDLINE, EMBASE, and the Cochrane library databases were searched up to June 2022 for randomized controlled trials that compared denosumab and oral bisphosphonates in the treatment of glucocorticoid-induced osteoporosis. The following outcomes were extracted for comparison: percentage change in bone mineral density from baseline at the lumbar spine, total hip, femoral neck, and ultra-distal radius; percentage change from baseline in serum concentration of bone turnover markers; and incidence of treatment-emergent adverse events.

**Results:**

Four randomized controlled trials involving 714 patients were included. The pooled results showed that denosumab was superior to bisphosphonates in improving bone mineral density in lumbar spine (mean difference (MD) 1.70; 95% confidence interval (CI) 1.11–2.30; *P* < 0.001) and ultra-distal radius (MD 0.87; 95% CI 0.29–1.45; *P* = 0.003), and in suppressing C-terminal telopeptide of type 1 collagen (MD -34.83; 95% CI -67.37--2.28; *P* = 0.04) and procollagen type 1 N-terminal propeptide (MD -14.29; 95% CI -23.65- -4.94; *P* = 0.003) at 12 months. No significant differences were found in percentage change in total hip or femoral neck bone mineral density at 12 months, or in the incidence of treatment-emergent adverse events or osteoporosis-related fracture.

**Conclusions:**

Compared with bisphosphonates, denosumab is superior in improving bone mineral density in lumbar spine and ultra-distal radius for glucocorticoid-induced osteoporosis. Further studies are needed to prove the efficacy of denosumab.

## Introduction

Glucocorticoids are widely used for common medical conditions including rheumatoid arthritis, asthma and chronic obstructive pulmonary disease. Glucocorticoid use has been the critical cause of secondary osteoporosis and drug-induced osteoporosis [1–3]. Long-term glucocorticoid use caused bone fractures in 30–50% of patients [2, 4]. To prevent development of glucocorticoid-induced osteoporosis (GIO) in patients with long-term steroid use is of great significance.

Bisphosphonates are currently the most commonly used drugs for the treatment of GIO [5–7]. Oral bisphosphonates have been proved effective to suppress loss of bone mineral density (BMD) in patients with GIO by suppressing osteolysis [8–11]. However, some patients are unable to use bisphosphonates because of side effects including renal impairments and acute-phase reactions. And bisphosphonates have therapy plateaus after 3 to 4 years in increasing BMD [12]. As an alternative, denosumab has been found to be effective in GIO patients. Denosumab was given subcutaneously 60 mg every 6 months. Denosumab has been proved to increase BMD effectively without therapy plateaus, and was associated with less renal impairments and acute-phase reactions [13–15].

Several studies have been conducted to compare the efficacy and safety between denosumab and bisphosphonates [16, 17]. However, limited information is available about the efficacy of denosumab for GIO. Evidence-based medicine is still lacking to prove the clinical results between denosumab and bisphosphonates for GIO. Therefore, we performed a systematic review and meta-analysis to compare the efficacy and safety between denosumab and oarl bisphosphonates for patients with GIO. A better understanding of advantages and disadvantages of denosumab and bisphosphonates for GIO may be gotten form this meta-analysis.

## Materials and methods

### Inclusion criteria

Studies included in the meta-analysis met the following criteria: (1) target population: patients with GIO, (2) intervention: denosumab versus bisphosphonates, (3) methodological criteria: randomized controlled trials (RCTs). Review articles, case series, and case reports were excluded. Studies that could not provide raw data on the mean or risk ratio were exluced.

### Search strategy

Databases include MEDLINE, EMBASE, Web of Science, and the Cochrane Collaboration Library up to June 2022 were searched for relevant studies. We used the search terms “denosumab”, “bisphosphonates”, “osteoporosis”, “glucocorticoid”, and “randomized controlled trial” with combinations of the operators “NOT”, “AND”, and “OR”. Two authors (L.J. and J.D.) screened the studies independently.

### Quality assessment

Quality of the included studies was assessed by two authors (L.J. and J.W.) independently. Disagreements between them were resolved after discussion with another author (L.L.). For the included RCTs, the 12 criteria and instructions recommended by the Cochrane Back Review Group [18] were used for quality assessment.

### Data extraction

Data was extracted from the included studies by two authors (L.J. and J.D.) independently. General characteristics of each study were collected: year of publication, author, sample size, study design, and duration of follow-up. The following outcomes were extracted from studies for comparison: percentage change in BMD from baseline at the lumbar spine, total hip, femoral neck, ultra-distal radius; percentage change from baseline in serum concentration of bone turnover markers including C-terminal telopeptide of type 1 collagen (CTX) and procollagen type 1 N-terminal propeptide (P1NP); and incidence of treatment-emergent adverse events (AEs), infection and osteoporosis-related fracture. Treatment adverse events (AEs) refer to any unfortunate medical event that occurs during the course of drug treatment and is not necessarily causally related to drug treatment. In this study, adverse events include back pain, arthralgia, hypertension, infection, headache, atypical femoral fracture, osteonecrosis of the jaw, malignancy, and so on.

### Statistical analysis

The meta-analysis was performed using Review Manager version 5.3 (Cochrane Collaboration). Continuous outcomes were presented in terms of mean difference (MD) and 95% confidence interval (CI); and dichotomous outcomes were presented in terms of risk ratio (RR) and 95% CI. Statistical heterogeneity among studies was assessed using the χ2 test. *P* < 0.10 or I^2^ > 50% indicated substantial heterogeneity. A fixed-effect model was used for data with low statistical heterogeneity; otherwise, a random-effects model was used. *P* < 0.05 indicated statistically significant difference.

## Results

### Literature search

**Fig. 1:**
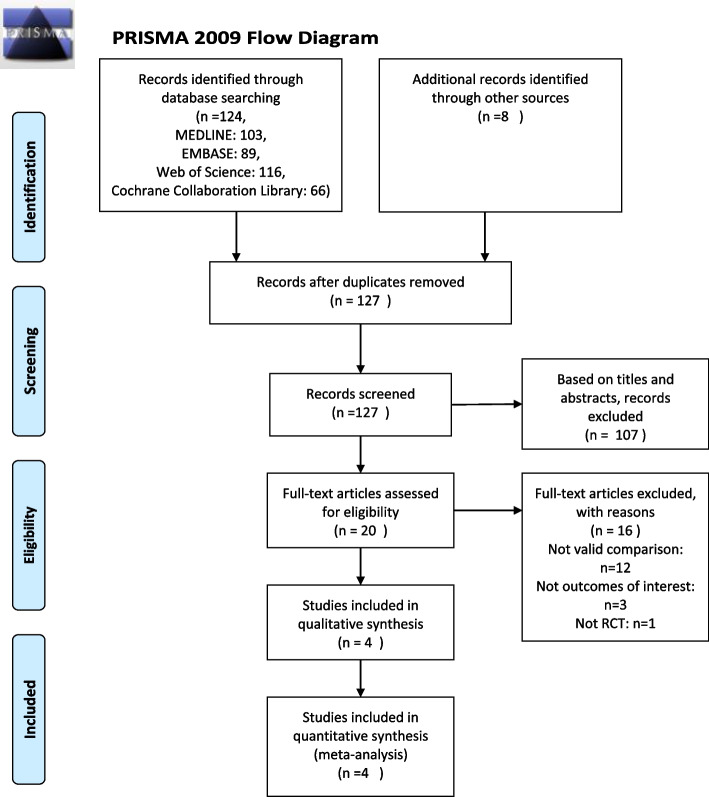
Flow of studies through review

The database search resulted in 132 articles that could potentially be included in the meta-analysis. One hundred twenty-eight articles were excluded after reviewing title, abstract, or full text. Finally, four RCTs [12, 16, 17, 19] were included in the study. Detailed steps of literature search are shown in Fig.1. The kappa score of the reviewers extracting the data was 0.78.

### Baseline characteristics

Four RCTs comparing the efficacy and safety of denosumab and bisphosphonates met the inclusion criteria. Sample sizes of the included four RCTs ranged from 28 to 505. Seven hundred fourteen patients including 357 in denosumab group and 357 in bisphosphonates group were included in the study. Patients in the two groups received denosumab 60 mg subcutaneously every 6 months, or oral bisphosphonates respectively. All the patients were given elemental calcium and calcitriol or vitamin D. Baseline characteristics of the two groups are shown in Table 1.

**Table 1:** Baseline characteristics of the studies included

Study	Treatment	Sample size	Age (year)	Female/male	Underlying disease (n)	Steroid dosage (mg/day) (prednisolone)	Steroid duration (months)	Follow-up (months)
Mok 2015 [17]	Denosumab	21	54.9 ± 12.8	21/0	Systemic lupus erythematosus (17) Rheumatoid arthritis (4)	4.60 ± 2.06	108.2 ± 56.0	12
	Bisphosphonates (unidentified)	21	54.6 ± 13.4	21/0	Systemic lupus erythematosus (15) Rheumatoid arthritis (6)	4.12 ± 2.14	94.1 ± 75.6	12
Iseri 2018 [18]	Denosumab	14	66.5 (39.0–75.8)	6/8	MCNS (4),Lupus nephritis (3), MN (2), ANCA-GN (3), FSGS (1), IgAN (1), HSPN (0)	5.0 (2.4–8.5)	82.8 (26.4–228)	12
	Alendronate	14	65.5 (45.0–78.5)	6/8	MCNS (5),Lupus nephritis (4), MN (3), ANCA-GN (0), FSGS (1), IgAN (0), HSPN (1)	5.0 (2.5–9.3)	108 (21.6–229.2)	12
Saag 2019 [12]	Denosumab	253	61.5 ± 11.6	185/68	Rheumatologic disorders (173), Respiratory disorders (46), Inflammatory bowel disease (3), Sarcoidosis (4), Neurologic disorders (11), Dermatologic disorders (9), Other (46)	12.3 ± 8.09	0–3: 5.1% 3–12: 32.0% ≥12: 62.5%	24
	Resedronate	252	61.3 ± 11.1	185/67	Rheumatologic disorders (184), Respiratory disorders (37), Inflammatory bowel disease (5), Sarcoidosis (5), Neurologic disorders (15), Dermatologic disorders (8), Other (37)	11.1 ± 7.69	0–3: 3.2%, 3–12: 29.8% ≥ 12: 66.3%	24
Mok 2021 [19]	Denosumab	69	52.0 ± 12.3	68/1	systemic lupus erythematosus (81%), rheumatoid arthritis (9.4%), inflammatory myopathies (5%), systemic vasculitis (3.8%)	5.1 ± 2.9	111 ± 62	12
	Alendronate	70	48.0 ± 12.9	65/5		5.0 ± 2.4	104 ± 69	12

*MCNS* minimal change nephrotic syndrome; *MN* membranous nephropathy; *ANCA-GN* antineutrophil cytoplasmic antibody-associated glomerulonephritis; *FSGS* focal segmental glomerulosclerosis; *IgAN* immunoglobulin A nephropathy; *HSPN* HenochScho ¨nlein purpura nephritis

### Quality assessment

**Fig. 2:**
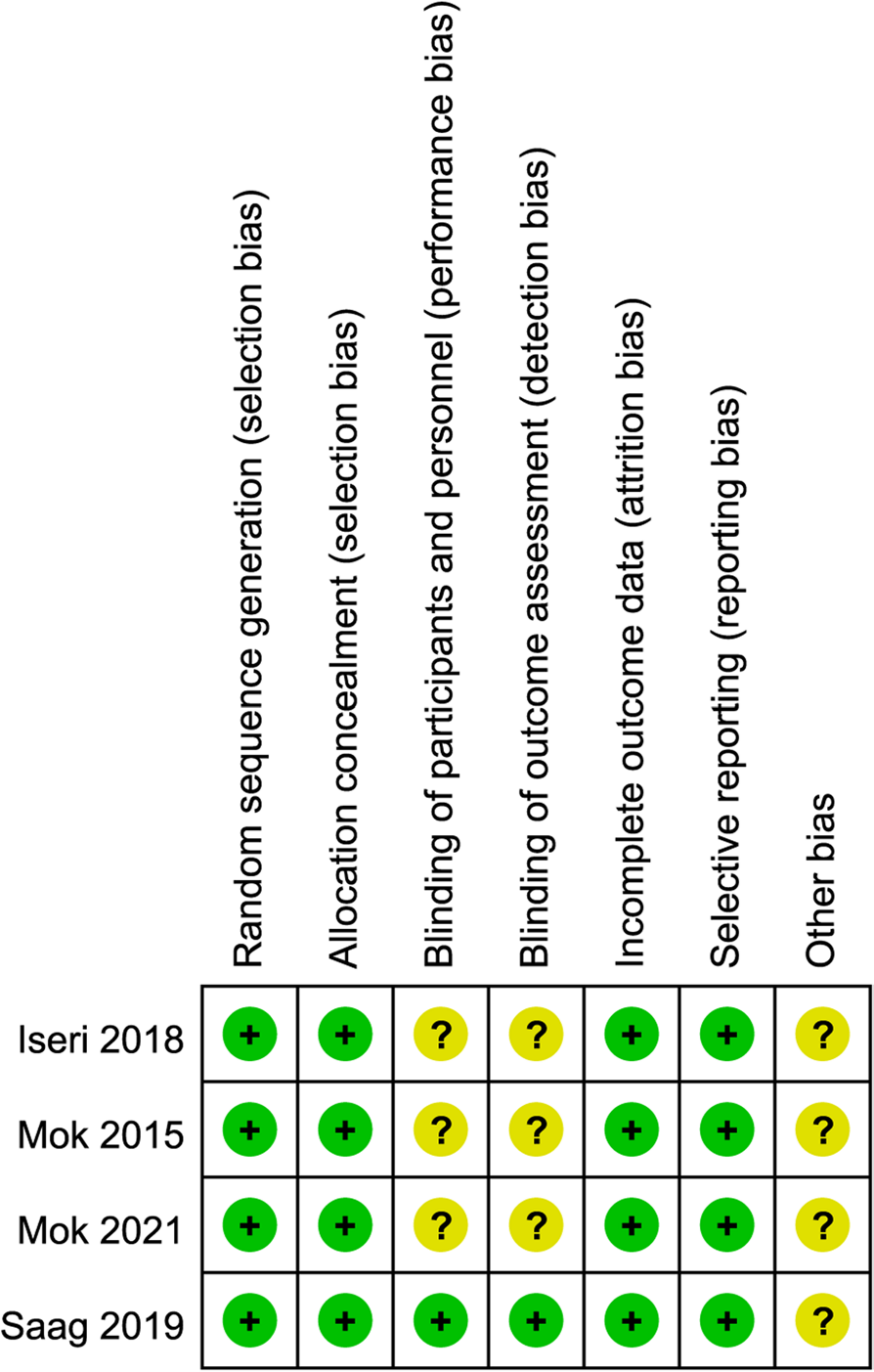
Quality assessment of the 4 studies included

All the included four studies used randomized design. The Cochrane assessment tool was used for quality assessment of the included studies. The final risk of bias of the included four studies was low based on quality assessment results. (Fig. 2).

### Clinical outcomes

**Fig. 3:**
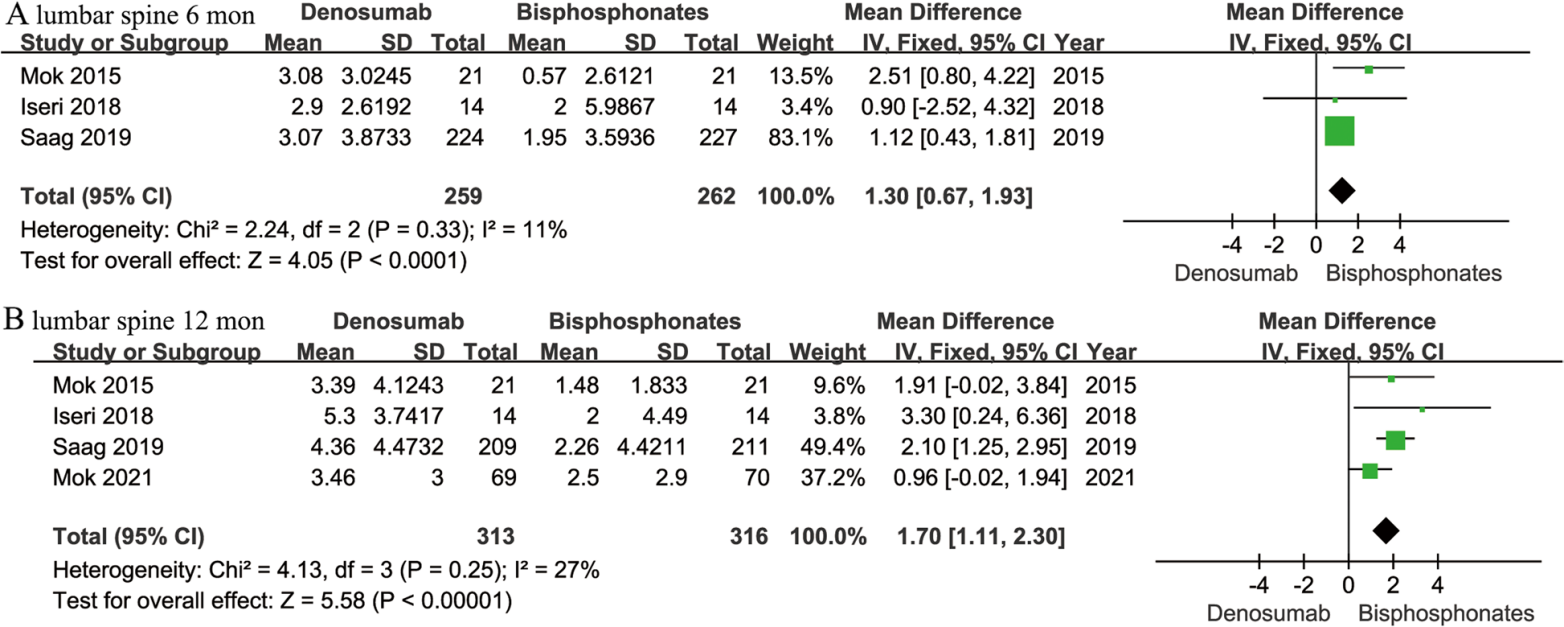
Forest plots of percentage changes in lumbar spine BMD at 6 months (**A**) and 12 months (**B**) in denosumab versus bisphosphonates

Percentage changes in lumbar spine BMD at 6 and 12 months were presented in three [12, 16, 17] and four [12, 16, 17, 19] studies respectively. It proved that denosumab was superior to bisphosphonates in increasing lumbar spine BMD at 6 (MD 1.30; 95% CI 0.67–1.93; *P* < 0.001) and 12 months (MD 1.70; 95% CI 1.11–2.30; *P* < 0.001) (Fig. 3).

**Fig. 4:**
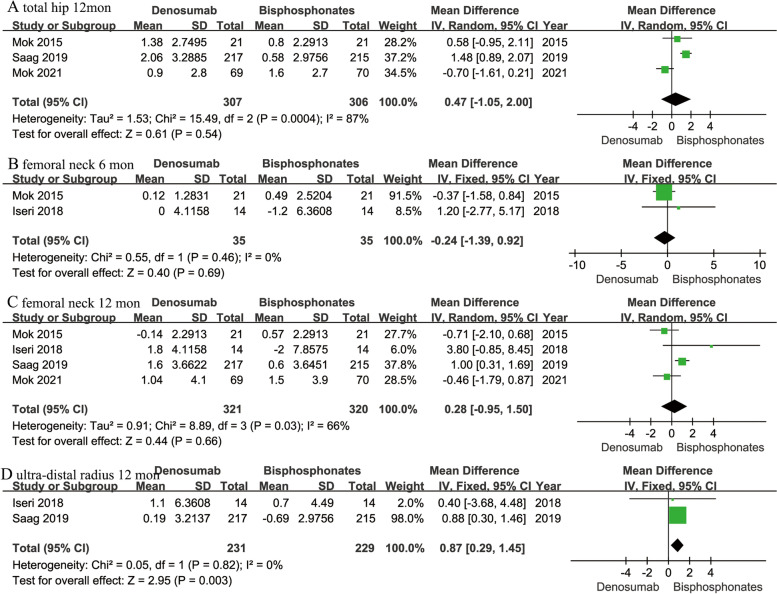
Forest plots of percentage changes of BMD in total hip at 12 months (**A**), femoral neck at 6 months (**B**) and 12 months (**C**), and ultra-distal radius at 12 months (**D**) in denosumab versus bisphosphonates

Percentage changes in femoral neck BMD at 6 and 12 months were recorded in two [16, 17] and four studies [12, 16, 17, 19] respectively. Pooled results showed there were no significant differences between two treatments at 6 months (MD -0.24; 95% CI -1.39-0.92; *P* = 0.69), or at 12 months (MD 0.28; 95% CI -0.95-1.50; *P* = 0.66) (Fig. 4).

Percentage changes of BMD in total hip and ultra-distal radius at 12 months were provided in three [12, 17, 19] and two studies [12, 16] respectively. The results showed the two treatments were similar in increasing total hip BMD (MD 0.47; 95% CI -1.05-2.00; *P* = 0.54), but denosumab was superior in increasing ultra-distal radius BMD than bisphosphonates (MD 0.87; 95% CI 0.29–1.45; *P* = 0.003) (Fig. 4).

**Fig. 5:**
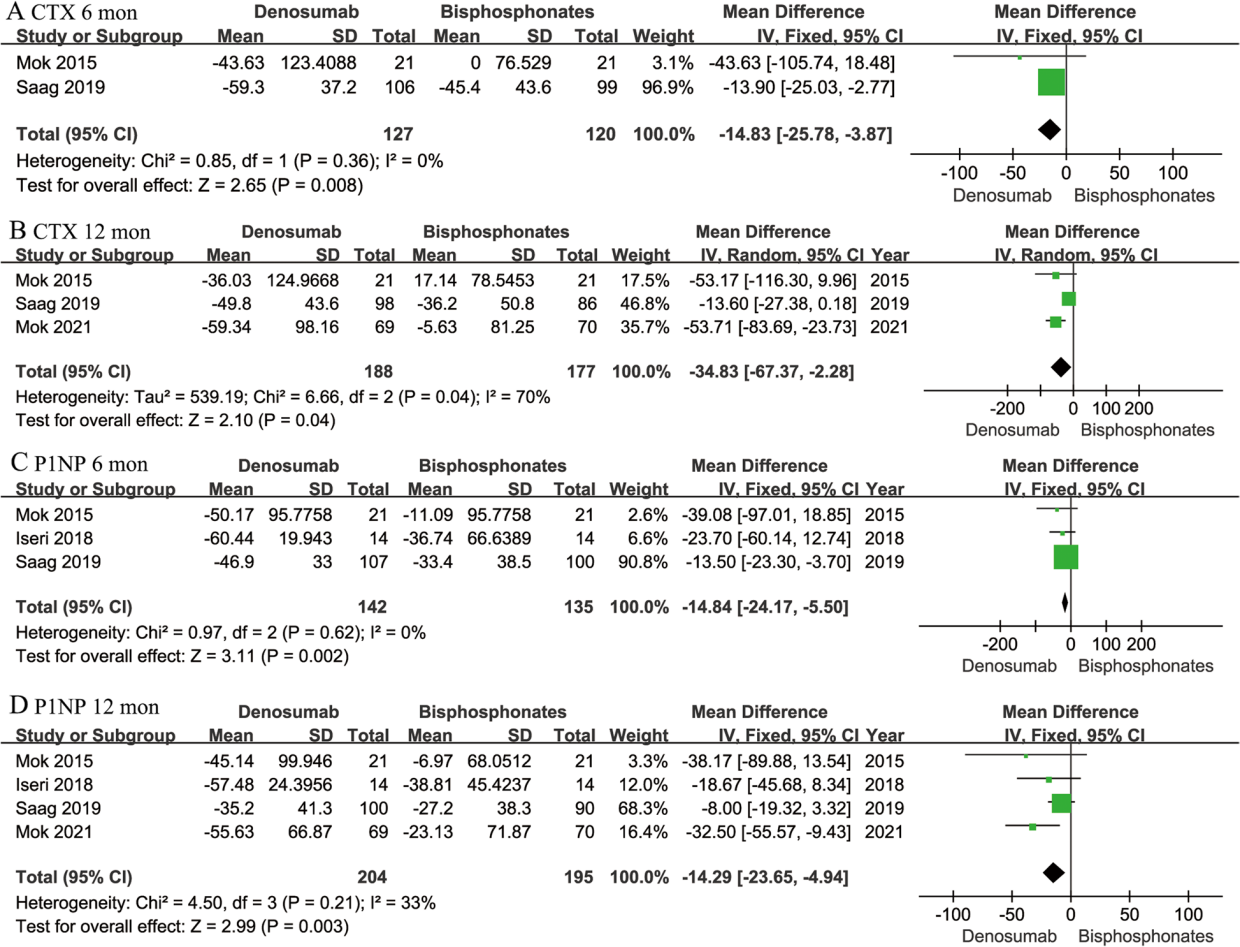
Forest plots of percentage change in serum CTX at 6 months (**A**) and 12 months (**B**), and P1NP at 6 months (**C**) and 12 months (**D**) in denosumab versus bisphosphonates

With respect to changes in marker of bone turnover, the percentage changes in serum CTX at 6 and 12 months were recorded in two [12, 17] and three studies [12, 17, 19] respectively. Pooled results showed denosumab was more potent than bisphosphonates in suppressing CTX at 6 months (MD -14.83; 95% CI -25.78- -3.87; *P* = 0.008), and at 12 months (MD -34.83; 95% CI -67.37--2.28; *P* = 0.04). The percentage changes in serum P1NP at 6 and 12 months were presented in three [12, 16, 17] and four studies [12, 16, 17, 19] respectively. Denosumab was associated with significant lower P1NP level at 6 months (MD -14.84; 95% CI -24.17- -5.50; *P* = 0.002) and at 12 months (MD -14.29; 95% CI -23.65- -4.94; *P* = 0.003) (Fig. 5).

**Fig. 6:**
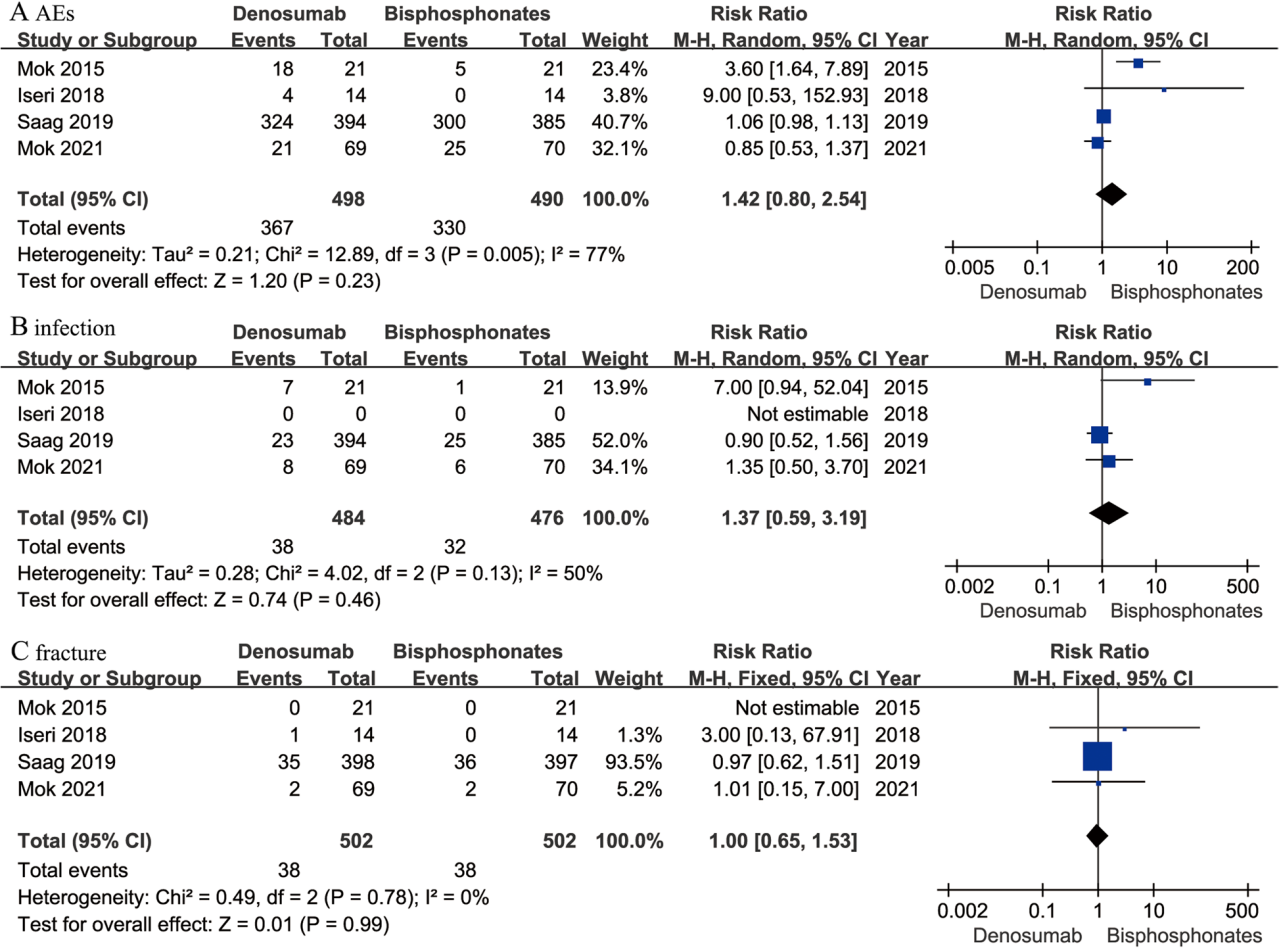
Forest plots of incidence of treatment-emergent AEs (**A**), infection (**B**), and osteoporosis-related fracture (**C**) in denosumab versus bisphosphonates

All the four studies [12, 16, 17, 19] recorded incidence of treatment-emergent AEs. The AEs included fracture, infection, skin rash, arthralgia, hypertension and so on. Pooled result showed no significant differences between two treatments in the incidence of AEs (RR 1.42; 95% CI 0.80–2.54; *P* = 0.23), infection (RR 1.37; 95% CI 0.59–3.19; *P* = 0.46) or osteoporosis-related fracture (RR 1.00; 95% CI 0.65–1.53; *P* = 0.99) (Fig. 6).

## Discussion

Both bisphosphonates and denosumab have been proved effective in rapidly decreasing bone resorption and increasing BMD. Bisphosphonates bind to the bone mineral, are taken up by the osteoclasts in the process of bone resorption, and inhibit the osteoclasts’ activity [20]. Denosumab is a human monoclonal antibody, binding to receptor activator of nuclear factor kappa-B ligand (RANKL) [21–23]. It neutralizes the function of RANKL which promotes osteoclastogenesis [20, 24]. As an alternative to bisphosphonates, denosumab has been found to be effective in the treatment of postmenopausal women with osteoporosis [25–28]. With respect to osteoporosis induced by glucocorticoid, several studies have been performed to compare the efficacy and safety between denosumab and bisphosphonates.

Based on the meta-analysis, denosumab is superior in improving lumbar spine BMD compared with bisphosphonates. However, there were no significant differences in total hip and femoral neck BMD improvement between two groups. It is clear denosumab is effective at improving BMD in trabecular bone [29]. The proportion of trabecular bone in the vertebrae is higher than that in the total hip or femoral neck, which may explain the different BMD improvements in lumbar spine, total hip and femoral neck after receiving denosumab treatment. Besides, in the comparisons of femoral neck and total hip BMD improvement at 12 months between two groups, there were substantial heterogeneity (I^2^ = 66%, I^2^ = 87%, respectively). Hence, more high-quality studies should be added for further evaluation in the future.

While denosumab has been proved effective in improving BMD in GIO patients, its discontinuation can lead to rapid bone loss and increased risk of so-called rebound-associated vertebral fractures [30]. So it’s important for patients to either continue denosumab, or receive other anti-resorptive drugs such as bisphosphonate to prevent the complication.

Regarding to incidence of treatment-emergent AEs, pooled result showed no significant differences between denosumab and bisphosphonates. One thing to note is that the follow-up period of the four included studies is relatively short, which may influence the final result. In previous studies comparing denosumab with zoledronic acid in advanced cancer with bone metastasis with long follow-up period, denosumab was associated with lower incidence of acute phase reaction, and renal toxicity, but higher incidence of hypocalcemia [31–34].

Based on this meta-analysis, there was no significant difference in fracture incidence between two treatment groups. However, it should be noted that the total number of fracture reported in the RCTs was low, and the RCTs were not powered to detect difference in fracture incidence between two groups.

There are several limitations of our study. First, the number of included studies is small. Second, all included studies were short in duration. Third, the patients included in the study had various backgrounds such as dosage of steroids, different types of bisphosphonates, and underlying diseases, which lead to significant heterogeneity. Fourth, influence of the study by Saag et al. [12] on the overall result of the meta-analysis is great, which may lead to bias.

In conclusion, for patients with GIO, denosumab is superior in improving BMD in lumbar spine and ultra-distal radius, and in suppressing CTX and P1NP than bisphosphonates. Future studies with larger sample size and longer follow-up period are advised to perform for further evaluation of denosumab.

## Data Availability

The data and materials contributing to this article may be made available upon request by sending an e-mail to the corresponding author.

## Abbreviations

GIO: Glucocorticoid-induced osteoporosis
BMD: Bone mineral density
RCTs: Randomized controlled trials
CTX: C-terminal telopeptide of type 1 collagen
P1NP: Procollagen type 1 N-terminal propeptide
AEs: Adverse events
MD: Mean difference
CI: Confidence interval
RR: Risk ratio
